# Influence of Electrospinning Parameters on Fiber Diameter and Mechanical Properties of Poly(3-Hydroxybutyrate) (PHB) and Polyanilines (PANI) Blends

**DOI:** 10.3390/polym8030097

**Published:** 2016-03-22

**Authors:** Ahmed M. El-hadi, Fatma Y. Al-Jabri

**Affiliations:** 1 Department of Physics, Faculty of Applied Science, Umm Al-Qura University, Al-Abidiyya, P.O. Box, 13174, Makkah 21955, Saudi Arabia; PHBhadi1963@yahoo.com; 2 Higher Institute of Engineering and Technology, Department of Basic Science, El Arish, North Sinai 9004, Egypt

**Keywords:** electrospun fiber, conductive biopolymers, poly(3-hydroxybutyrate) (PHB), Polyanilines (PANI), mechanical properties

## Abstract

Random and oriented fibers of poly (3-hydroxybutyrate) (PHB) and their blends were manufactured using electrospinning using a co-solvent. The kind and the concentration of the co-solvent affected the diameter of electrospun fibers. The morphology, thermal analysis, and crystalline structure of electrospun fibers was studied using polarized optical microscop (POM), Differential scanning colametry (DSC), Scanning Electron Microscopy (SEM), Wide angle X-ray diffraction (WAXD), and FT-IR analysis. The diameter of the electrospun fibers decreases with increasing collector speed for the blends compared to pure PHB, which are about 6 µm in diameter. The fibers obtained from blends reduce to 2 µm. The aligned electrospun fiber mats obtained from pure PHB showed no signs of necking at different take-up speeds, but the blends show multiple necking. It was found by FT-IR that the peak intensity at 1379 cm^−1^ was lower by take up speed than in casting films; this peak is sensitive to crystallinity of PHB. The addition of polyanilines (PANIs) to (PHB) with a plasticizer decreases the diameter of the electrospun fiber.

## 1. Introduction

Electrospinning is a process that produces polymer fibers with diameters in the range between micrometer to nanometer dimensions with an applied high voltage. Higher electrical power is used to overcome the strength of the surface tension on the surface of the polymer solution, and a very thin charged jet is created.

The jet forms a straight line, and the electrical forces elongate the jet 1000 times, making it very thin. In electrospinning, when an electrical voltage is applied between two electrodes, the droplets of liquid that are in the end of the needle form the so-called Taylor cone. The processing parameters that control the formation of the fiber are the solvent evaporation, the flow rate, the internal needle diameter, the distance from the needle to the collector, and the applied voltage [1].

In recent years many scientists have shown great attention to the use of electrospun PHB fibers in medicine to replace damaged or poorly grown nerves and tissue regeneration. PHB are natural polymers formed by using bacteria during fermentation. PHB is one group of polyhydroxyalkanoates (PHAs) that can be manufactured in larger quantities. PHB is a biopolymer, thermoplastic, non-toxic, has high crystallinity, and its physical properties are similar to polypropylene [2,3]. PHB can be applied in biomedical requests, like surgical suture and wound dressings. However, the brittleness of PHB hinders this application [4].

PANI has good stability, good electrical conductivity, is biocompatible, and has low cost. PANI is difficult to dissolve in many solvents, so, it has poor processability. There is wide interest to develop biodegradable PHB, PANI, and plasticizers in the biomedical field to repair damaged nerves. Electrospun PHB fiber blends can be used in applications such as purification of water or air [5], biosensors [6], tissue engineering, wound healing, and release of drugs [7]. It can improve the physical properties of polymeric materials in many ways like a copolymers [8,9] or mixing the polymer with other polymers or addition plasticizers and lubricants [10,11,12,13]. The main goal is to preserve the main properties of the basic material with solving its disadvantages for use in the industry, while taking the economical approach and biodegradation process approach. The blend consists of two different polymers. Most of the polymers are classified into completely immiscible [14], partially miscible, or completely miscible. It is well known that the completely miscible blend [15] can improve the thermal and mechanical properties and biodegradation than another immiscible mixture [16]. In polymeric systems blends (full mixing) result in one glass transition temperature, where the interactions between the particles are stronger, like ionic bonds, dipole-dipole interactions, or hydrogen bonds [17,18]. The immiscible blends form two or more glass transition temperatures and the interactions between the particles is weaker, *i.e.*, all mixing polymers components are in phase separation and each material keeps with its individual characteristics.

Components that are miscible in the case of the solution, or molten state, but are separate structures in the solid state phase, include poly(3-hydroxybutyrate) and polyethylene oxide PHB/PEO [1,19], (poly(3-hydroxybutyrate)/poly(vinylidene fluoride) PHB/PVDF blend [20], and (poly(3-hydroxybutyrate)/poly(l-lactic acid) PHB/PLLA blend [21]. In addition, in the case of a miscible polymer blend by co-solvent, the resulting miscible mixture depends greatly on many factors, such as single solubility of polymers in a number of solvents, and the concentration of the polymer solution and the evaporation rate of the solvent. It is possible to get non-miscible or miscible polymer blends from the some polymers but not the same solvents. In this study, electrospun PHB fibers and their blends were successfully fabricated by electrospinning using different solvents. The main objective of this work is to manufacture (1) wound dressings of PHB with plasticizer; (2) the addition of PANIs + PHB + plasticizer to make material as conductive biopolymer fiber. The fiber can be used for the transfer the electrical signals from the brain to nerve system (replacing damaged nerves as a result of an accident or war) [1,22].

The aim of this work is to investigate the effect of electrospinning factors, such as applied voltage, inner needle diameter, and flow rate of the solution on fiber morphology. The structure and mechanical properties of the obtained fibers was analyzed by a tensile testing device and scanning electron microscopy (SEM).

## 2. Materials and Methods

### 2.1. Materials

The molecular weight of PHB used is *M*_W_ = 2.3 × 10^5^ g·mol^−1^. The other materials used were PANI polyaniline salt and a plasticizer (tributyl *O*-acetylcitrate 98%), and solvents, such as chloroform (CF), dichloromethane (DCM), and dimethylformamide (DMF). All materials were purchased from Sigma-Aldrich, Germany.

#### 2.1.1. Sample Preparation

At first, we mixed PHB (80%) with a plasticizer (20%) in chloroform and left to dry to form a cast film, then dissolved it again with PANIs using a mixture of solvents (90% chloroform + 10% DMF or chloroform 75% and 25% dichloromethane). The investigated PHB/plasticizer/PANIs blends had weight ratios as follows: pure PHB (100/0/0), blend 1(80/20/0), blend 2 (79/20/1), blend 3 (77.5/20/2.5), blend 4 (75/20/4), and were prepared by a solution method.

#### 2.1.2. Electrospinning Equipment

PHB and its blends dissolved in different solvents at different concentrations and placed in a plastic syringe (5 mL) that connected to a needle with an inner diameter (ID) of 1.3 mm. Electrospinning was performed at room temperature with a home-made take-up rotating drum with variable speed and a high-voltage power supply from USA (model NO. ES60P-20W, Gamma High Voltage Research, Orlando, FL, USA). All fibers were pure PHB and their blends collected on the aluminum foil. A syringe-pump (No. BS-9000-USA, Braintree Scientific, Braintree, MA, USA) was used to feed the polymer solutions into the needle tip. The electrospun fibers were collected on a grounded collecting plate or rotating drum (homemade) with a maximum speed of 1100 rpm.

### 2.2. Sample Characterization

#### 2.2.1. Thermal Analysis

The thermal analysis of PHB and its blend were calculated by NETZSCH DSC 204 F1 Phoenix under a nitrogen atmosphere. The samples (about 5 mg weight) were heated from 25 °C to 200 °C, held for 1 min, and then quenched to −50 °C; the endo- and exothermal curves were then recorded. The glass transition temperature (*T*_g_) was obtained as the variation point of the heat capacity with a heating rate of 10 °C/min and temperature −50 to 200 °C.

#### 2.2.2. Wide Angle X-ray Diffraction (WAXD)

Wide angle X-ray diffraction (WAXD). The measurements were carried out using a Panalytical X’pert PRO diffractometer (PANalytical B.V., Eindhoven, The Netherland) with Ni-filtered Cu Kα radiation with a wavelength of λ = 1.54178 Å over the 2θ range of 5–35° at 40 kV. WAXD data for pure PHB and their blends with additives were obtained at room temperature (~25 °C) with a scan rate of 2° 2θ min^−1^. Solution cast film samples were cut into rectangular pieces (4 cm^2^) and mounted on the matrix prior to analysis.

#### 2.2.3. FT-IR Spectroscopy

Infrared spectra of the investigated films, which were cut into rectangular pieces of 4 cm^2^ area, were recorded at room temperature and over the wavenumber range 550–4000 cm^−1^ using a Fourier Transform FT-IR 6100 Jasco (Jasco analytic instrument, Tokyo, Japan) spectrometer.

#### 2.2.4. Scanning Electron Microscopy (SEM)

The surface morphology of the PHB nanofibers and their blends was observed using a scanning electron microscope (JSM-6360LA, JEOL Co., Boston, MA, USA) at an accelerating voltage of 15 kV. The samples were sputter-coated with gold for 120 s to a thickness of 2–3 nm using a sputter coater (EMITECH K550X, Kent, UK). Images of several sample fibers were obtained using SEM to measure the fiber diameter. The samples are coated with gold.

#### 2.2.5. Mechanical Properties

Uniaxial tensile tests were made with dog-bone specimens (width 4.8 mm, length 22.25 mm, thickness 15–35 μm). All samples were tested in the tensile test device and cut in a dumbbell shape (Dumb Bell Ltd. SDL-100, DUMBBELL Co. Ltd., Saitama-Ken, Japan). Sample thickness was measured using a micrometer. Tensile tests were performed at room temperature with speed of 1 mm·min^−1^, using a Shimadzu universal testing machine equipped with a 10 kN load cell and connected with a computer. Five specimens of each formulation were tested and the average values are reported. From the correlation between stress σ (in Pa) and elongation ε (in %), *ε* = (*L*_0_ − *L)/L*, *L*_0_ = original length, *L* = length after elongation. At the end, the fracture surface of the electrospun samples was studied by SEM.

## 3. Results and Discussion

### 3.1. Fiber Morphology by Scanning Electron Microscopy (SEM)

**Figure 1:**
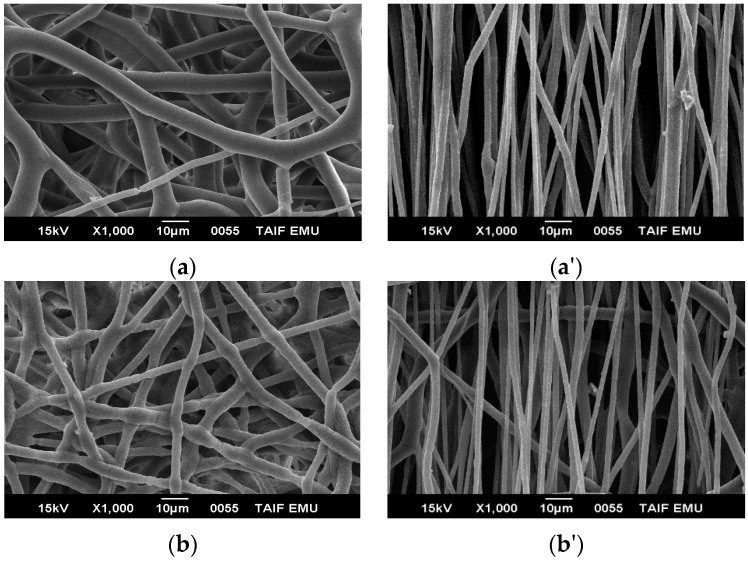
The samples of pure PHB and blend 1 were collected on the aluminum foil with the following die diameter, applied voltages, CF/DCM ratios, flow rate, and the distance between needle and target: 20 wt %, 1.3 mm, 20 kV, 75:25, 0.185 µm·h^−1^, and 20 cm, respectively; (**a**,**a'**) pure PHB at a fixed target (random) and rotating drum with a speed of 1100 rpm with polymer concentrations of 20%; (**b**,**b'**) blend 1 at a fixed target (random) and at a rotating drum with a speed 1100 rpm with polymer concentrations of 25%.

SEM was performed after using the information from POM on how to get the best electrospun fibers and select the best parameters determined previously. The SEM images show that the electrospun fiber diameters are in the range of micrometers. Figure 1a,a' shows the morphologies of pure PHB electrospun fiber mats. After the solvent evaporates, fibers with a submicron-level diameter are formed on a collecting plate or rotating collector at different speeds. It is easy to deform the fibers by random or rotation spinning, which implies that the fiber is elastic and flexible. By using the rotating collector, the fiber can be oriented (aligned). The fibers were formed without beads, and the average fiber diameter was ~5 μm. We can see the orientation of the fibers upon using different speeds by the rotating drum. A necking region was not observed in pure PHB electrospun fibers that were collected at the fixed plate (random) or the rotation drum (aligned).

**Figure 2:**
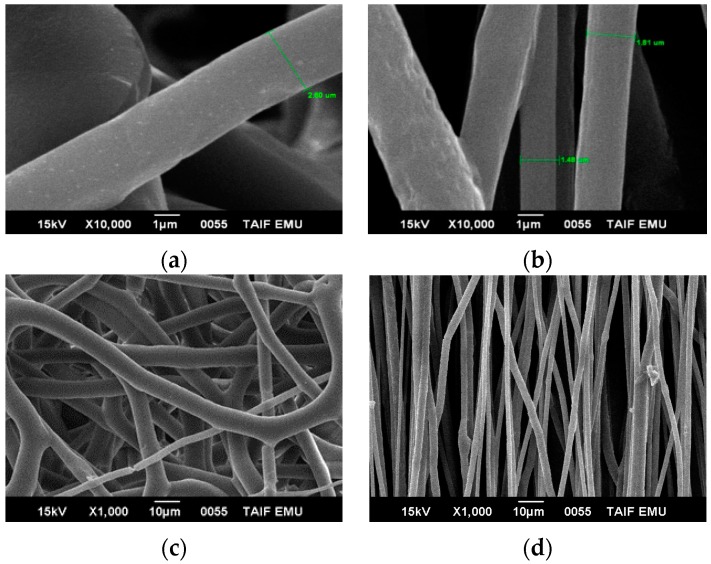
The samples of pure PHB at different scales were collected on the aluminum foil with the following polymer concentrations, die diameter, applied voltages, CF/DMF ratios, flow rate, and distance between the needle and target: 20 wt %, 1.3 mm, 20 kV, 90:10, 0.185 µm·h^−1^, and 20 cm, respectively. (**a**,**c**) at a fixed target (random) with different scales, and (**b**,**d**) at a rotating drum with a speed of 1100 rpm.

Figure 1b,b' show the SEM images of blend 1 electrospun fiber mats that were collected using a fixed plate and rotating collector at different speeds. It is clear that the fibers appear smooth and that the alignment of the fibers was enhanced at higher speed, *i.e.*, upon increasing the take-up speed, there is good alignment and the fiber diameter reduces. Figure 2 shows pure PHB electrospun fiber mats with an average fiber diameter ~2 µm. It was found that, with increasing the collector speed, the fiber diameter reduces.

Figure 2 shows the morphology of the electrospun fiber mats of pure PHB. Pure PHB have diameters between 1.48 to 2.5 µm. It is manufactured by stretching the fiber using a rotating drum at 1100 rpm. The aligned electrospun fibers of pure PHB and showed no signs of necking during take-up at higher speed rotation compared with lower speed*.* Therefore, we use CF/DMF to obtain thinner fiber compared with using CF/DCM in the same sample. The average diameter of the fibers decreased significantly, and beads were not detected in pure PHB.

**Figure 3:**
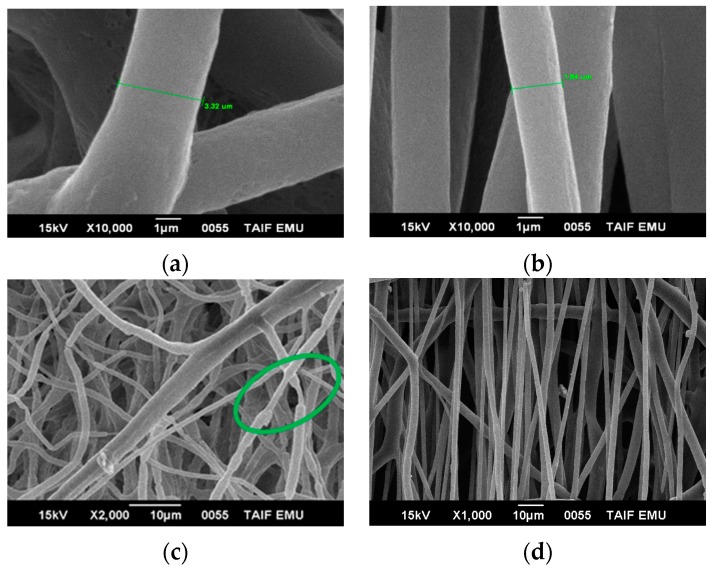
The samples of blend 1 at different scales and speeds were collected on the aluminum foil with the following polymer concentrations, die diameter, applied voltages, CF/DMF ratios, flow rate, and distance between the needle and target: 25 wt %, 1.3 mm, 20 kV, 90:10, 0.185 µm·h^−1^ and 20 cm, (**a**,**c**) at fixed target (random); (**b**,**d**) at a rotating drum with a speed of 1100 rpm, respectively.

Figure 3 shows blend 1 electrospun mats with average fiber diameters of ~2 µm*,* (using CF/DMF and rotating speed 1100 rpm). Therefore, we use CF/DMF to obtain a thinner fiber compared with using CF/DCM in the same sample. The average diameter of the fibers decreased significantly. Beads were not detected in pure PHB and both the blends use the co-solvent CF/DMF.

The aligned electrospun fibers of pure PHB (Figure 2) and blend 1 showed no signs of necking during take-up at higher speed rotation compared with lower speeds. The average electrospun fiber diameters of blend 1 were between 2 and 2.5 µm. We compared the randomly-oriented fibers in the same sample, which are about 3 µm in diameter, but the fibers obtained here have about two times smaller diameter. The decreased diameter of the electrospun fibers of PHB and their blends was attributed to higher take-up speed and the conductivity of DMF solvent. Fibers of pure PHB and blend 1 showed smooth surfaces. No necking region was observed in the fibers collected on the rotating drum (aligned fibers), *i.e.*, the average diameter of the fiber was smaller at higher take-up speed if the fibers were spun using the same polymer at lower take-up speed.

**Figure 4:**
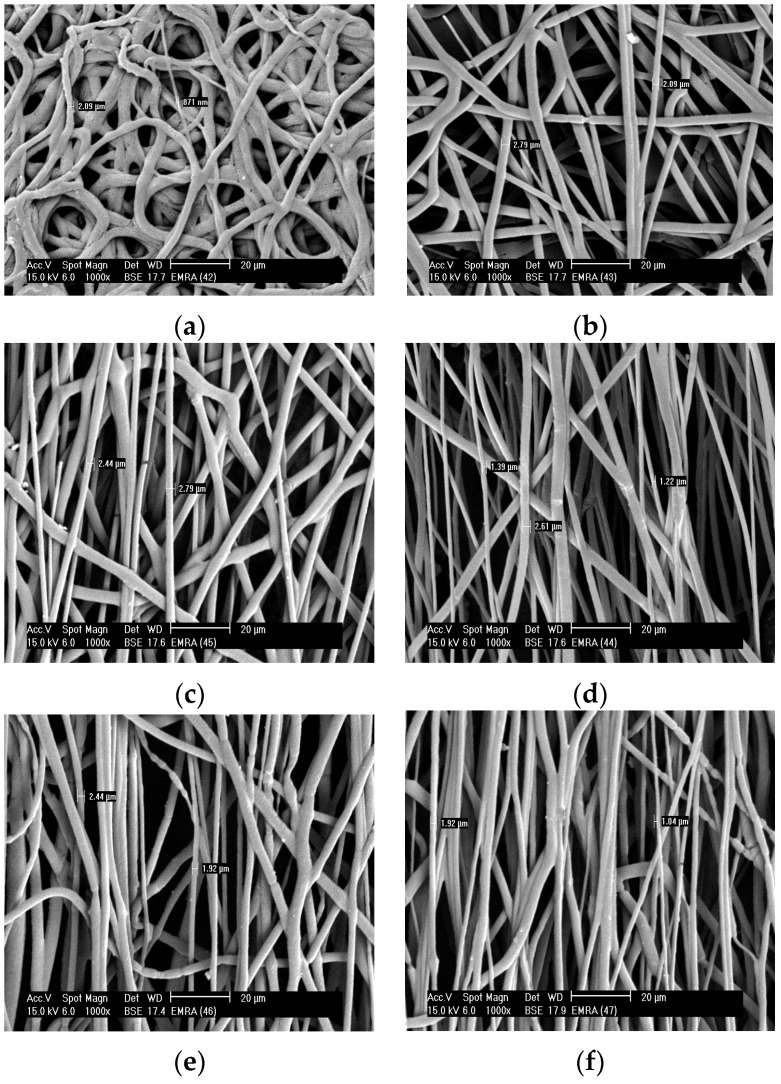
The samples of blend 2 were collected on the aluminum foil with the following polymer concentrations, die diameter, applied voltages, CF/DMF ratios, flow rate, and distance between the needle and target: 25 wt %, 1.3 mm, 20 kV, 90:10, 0.185 µm·h^−1^, and 20 cm. (**a**) at a fixed target (random); (**b**) at a rotating drum with a speed of 380 rpm; (**c**) at a rotating drum with a speed of 490 rpm; (**d**) at a rotating drum with a speed of 610 rpm; (**e**) at a rotating drum with a speed of 740 rpm, and (**f**) at a rotating drum with speed 850 rpm.

Figure 4 shows certain necking regions in electrospun fibers by collecting random and aligned fibers of blend 1 (Figure 3) and blend 2 (Figure 4). Electrospun fibers obtained from blend 2 were much thinner than the fibers obtained from pure PHB and blend 1 by using the same co-solvent, CF/DMF. Smooth blend 2 fibers are formed with diameters between 0.87 and 2.5 µm, approximately. In many uses it is necessary to develop aligned fibers. By increasing the take-up speed, thinner fibers can be formed. The fibers were fully toughened before they were collected and had a smooth texture. PHB fibers show no necking regions; however, PHB blends 1 and 2 show a developed fiber neck at both random and rotation spinning. All electrospun fibers showed smooth surfaces. The existence of a necking region means that the fiber is not able to resist the mechanical or electrical pull which would lead to plastic deformation in different positions along the fiber, *i.e.*, the fibers are more elastics, flexible, it can deformat at higher speed.

### 3.2. Mechanical Properties

The mechanical properties of PHB and PANIs can be improved by the addition of a plasticizer. Flexible elastic films or electrospun fibers with a significant reduction in stress and increased elongation can be obtained. It is known that PHB is brittle and rigid [2,3] and its elongation at break (4%) [3], by using the rotation drum at a speed of 610 rpm; PHB did not give good elongation.

**Figure 5:**
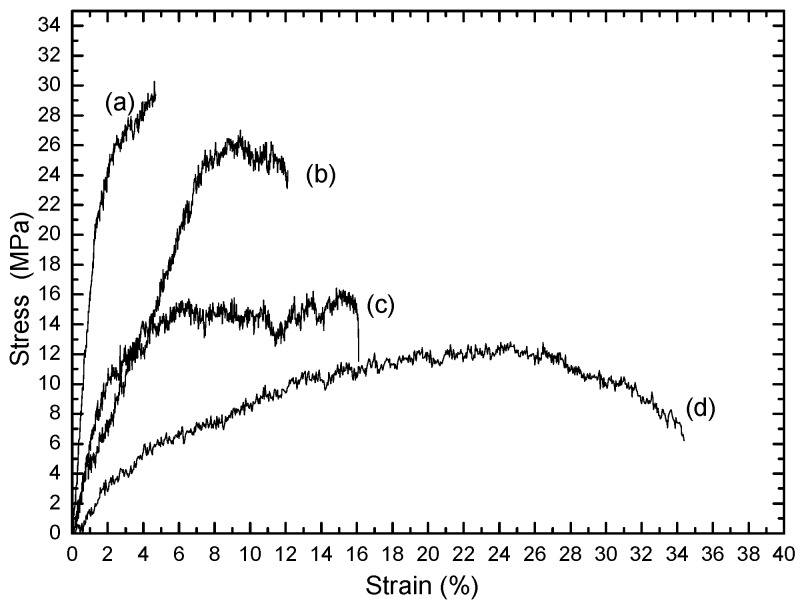
Stress-strain curves of nanofibers: (**a**) pure PHB with a speed of 610 rpm; (**b**) blend 2 with a speed of 610 rpm; (**c**) blend 2 (random); and (**d**) blend 1 (random).

Figure 5 shows the stress–strain curves for electrospun fibers of pure PHB, blend 1, and blend 2. The tensile stress decreases from 29 MPa for pure PHB to 26 MPa for blend 2 using a take-up speed of 640 rpm, 16 MPa for blend 2 (random), and 10 MPa for blend 1 (random). The results show that the addition of 20 wt % plasticizer has a effect on the mechanical properties of the electrospun fibers of PHB. The elongation at break increased to 34%, while the tensile strength decreased to 10 MPa. The addition of PANIs to PHB with plasticizer decreases the elongation at break to 15% and increases the stress to 16 MPa. The presence of PANIs in blend 2 increases the tensile strength, enabling the use of the material in different applications.

Figure 5 shows electrospun fibers of blend 2 spun at a high take-up velocity of 610 rpm, which demonstrated high tensile strength and lower strain compared to electrospun fibers of the same sample collected at a fixed target (random fibers).

**Figure 6:**
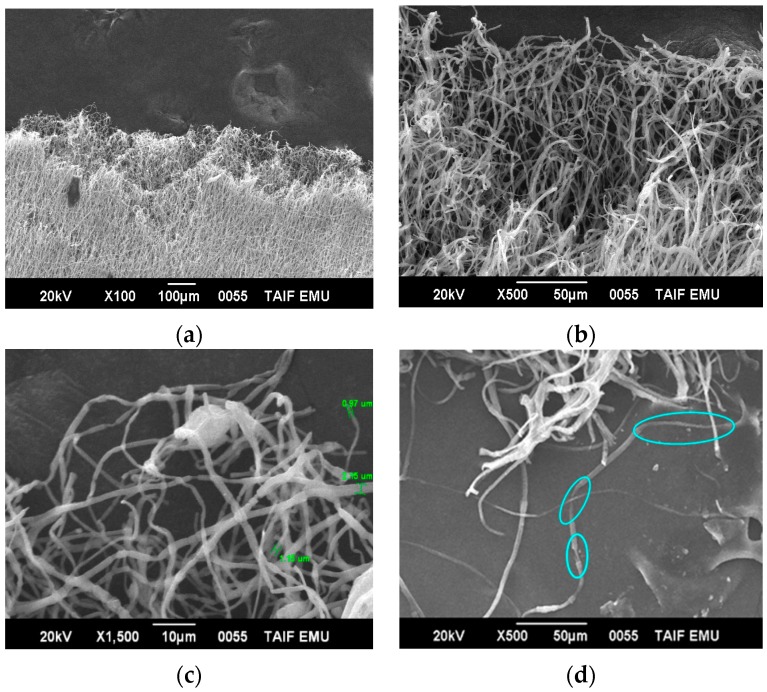
SEM micrographs of fractured surfaces of multiple neck formations in electrospun fibers of blend 2 (**a**, **b**, and **c**) and blend 1 (**d**) after cold drawing, at different magnifications.

Figure 6 shows the tensile tests after cold drawing (destroyed surfaces) of the electrospun fiber of blends 1 and 2 using SEM analysis. It has been observed that the electrospun fibers have multiple necking. In addition, multiple necking of the electrospun fibers happens along the fiber, which would lead to the change of a fibril structure as shown in Figure 6 at a high magnification. Various necking takes place only by collecting blends 1 and 2 at the fixed target (random). Small necking regions of around 0.97, 1.15, and 2.15 μm were observed along each fiber. The mechanical behavior, in general, depends on the fiber diameter. The electrospun fibers deformed via wide-ranging necking and plastic deformation before breaking after cold drawing, as presented in Figure 6c,d.

When the tension was increased, the necking regions were transmitted homogeneously along the fibers until the complete fiber mat broke (Figure 6c,d). The formation of the necks led to a decrease in the fiber cross-sectional area. The fibers have some smaller neck regions that appear at the random collector or by tensile testing with a different necking length. Necking transmits along the fiber until the fiber completely fails. 

### 3.3. Thermal Analysis (DSC)

DSC analyses were used to determine the melting and crystallization temperatures of PHB and its blends with PANIs and plasticizer. Figure 6a shows the DSC curves of the first heating, Figure 6b for cooling, and Figure 6c for the second heating. In the first heating curves, it is observed that pure PHB and its blend with plasticizer showed a melting temperature at approximately 151 and 169 °C. Two melting temperatures are also detected on PHB and its blend.

**Figure 7:**
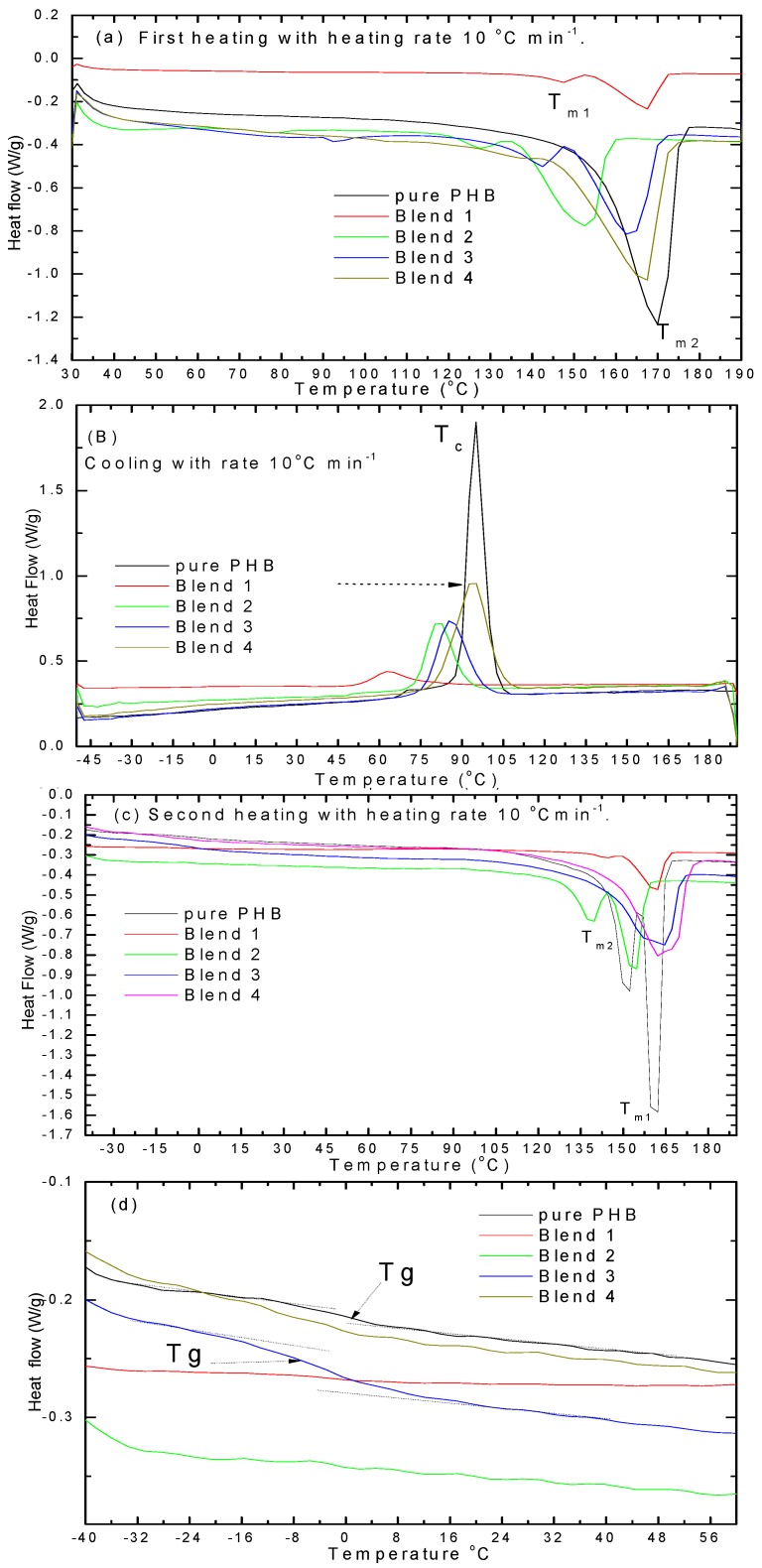
DSC curves for pure PHB and its blends (**a**) first heating; (**b**) cooling; (**c**) second heating; and (**d**) second heating to determine the glass transition temperature region.

These double peaks are attributed to two lamellar thicknesses with different crystal structures (the smallest melted first and the largest melted second). The melting temperatures of PHB with plasticizer were lower than that for pure PHB. It was observed that the plasticizer has an effect on the *T*_c_ and *T*_m_ during the first heating, cooling, and second heating curves. By cooling, the *T*_c_ is shifted from 95 °C for PHB to 63 °C for blend 1, the T_c_ shifted to 81 °C for blend 2, 85 °C for blend 3, and 94 °C for blend 4. Upon second heating there is no recrystallization peak for both PHB and its blends. There is no cooled crystallization temperature (*T*_cc_) observed because the crystallization occurred during cooling. This result shows that PANIs acted as nucleating agents. However, an improvement in crystallization was found for blend 4 with 4 wt % PANIs. The second melting temperature was shifted slightly to higher temperatures compared to the first heating for blends 1, 3, and 4. This occurred because the crystallization conditions are different than the first preparation of the film. It is known that the glass transition temperature *T*_g_ from DSC is around 2–5 °C [1,2] for PHB. It is clear from Figure 7d that there is one (*T*_g_) for all blends with PANIs as few ratio between 1% and 4%, meaning that it's a good mixed (miscible). For this reason, we concluded that all blends were miscible systems. However, the *T*_g_ of PHB in blends preserve unchanged with weight fraction (1%–4%) of the PANIs at about −7 °C. The PANIs can only be affected the crystallization process and serves as a nucleation agent.

### 3.4. WAXD Analysis

**Figure 8:**
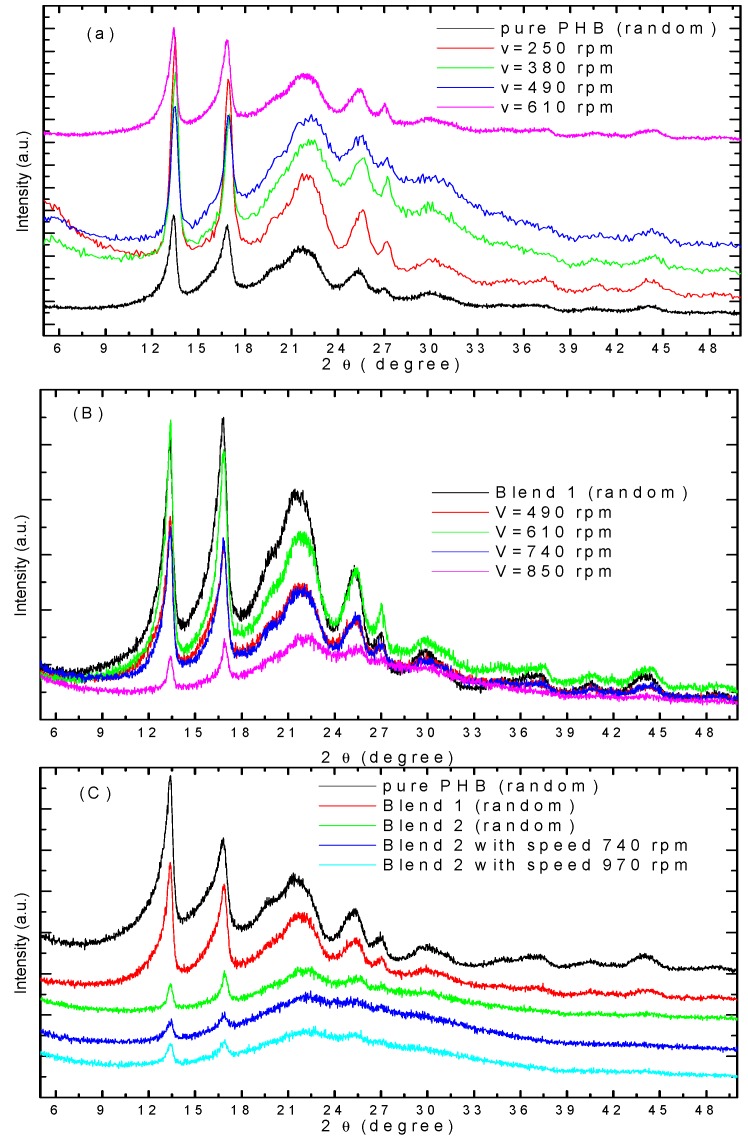
WAXD of electrospun fibers of (**a**) pure PHB with different take-up speeds (v), (**b**) blend 1 with different take-up speeds, and (**c**) pure PHB, blends 1, 2, and blend 2 with different take-up speed (v).

Figure 8 shows the WAXD of electrospun fibers of pure PHB and their blends with different take-up speeds. The characteristic reflection peaks of crystalline PHB at 2θ = 13.6 and 16.9° indicates the formation of PHB crystalline phase in the pure sample [1,2]. However, a certain reduction of the main reflection peaks at 2θ = 16.9° and some orientation at 2θ = 13.6°, as shown in Figure 8a, occurs because the crystalline structure of PHB deformed through the electrospinning process at higher take-up speeds. Similar results occur in blend 1 with different take-up speeds, as shown in Figure 8b. Figure 8c shows the WAXD peaks of electrospun of pure PHB (random), blend 1 (random), blend 2 (random), and blend 2 with different take-up speeds. The intensities of the crystalline peaks were reduced by addition of different PANI content and by varying the take-up speeds. The electrospun fiber shows a broad pattern with small crystalline structures, which implies that the crystals in the fibers are not well developed. In the cold drawing process, which includes the stretching of electrospun fibers at a higher velocity, a broadening of the peak was observed in the electrospun fiber sample (blend 2), indicating a stretching and change in the structure during the electrospinning process. This broad peak is of non-crystalline PANI.

### 3.5. FT-IR Analysis

**Figure 9:**
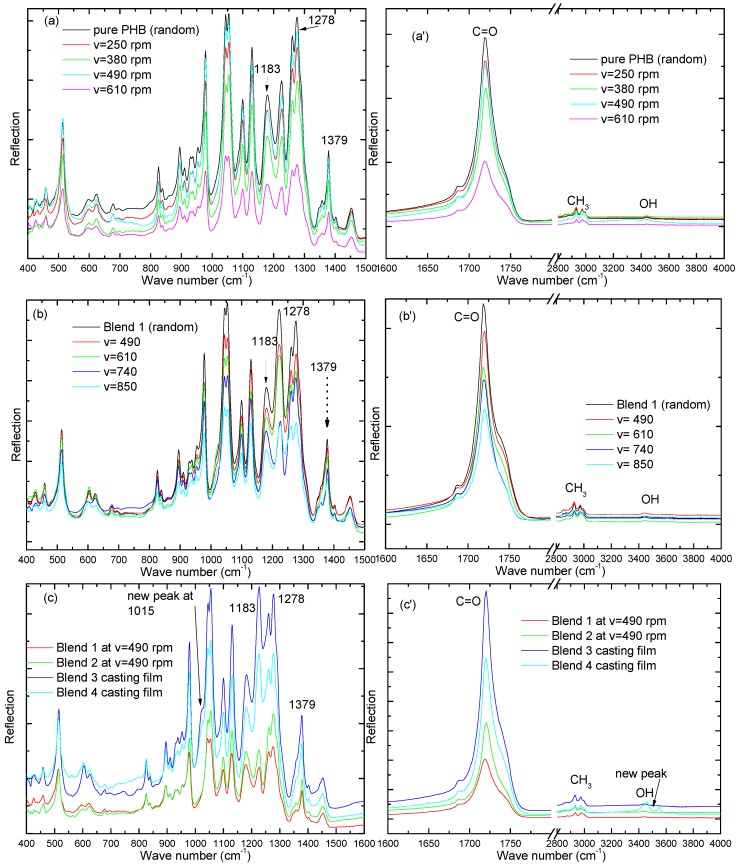
FT-IR spectrum of electrospun PHB and their blends in the infrared spectrum regions at different take-up speeds (**a**) from 400 to 1600 cm^−1^ of PHB and (**a'**) from 1600 to 4000 cm^−1^ of PHB; (**b**) from 400 to 1600 cm^−1^ of blend 10; and (**b'**) from 1600 to 4000 cm^−1^ of blend 10; (**c**) from 400 to 1600 cm^−1^ of blends 10, 14, 16, 17, and (**c'**) from 1600 to 4000 cm^−1^ of pure PHB and blends 1 and 2, at constant take-up speeds of 490 rpm, but blends 3 and 4 as a casting film.

The FT-IR spectrum of electrospun PHB fibers and their blends are shown in Figure 9 at different take-up speeds. They show a series of characteristic bands that are split into three bands around 1686, 1723, and 1742 cm^−1^, which correspond to the ester carbonyl group (1723 and 1686 cm^−1^ crystalline and 1742 cm^−1^ amorphous). The main characteristic bands at 1278 cm^−1^ correspond to the –CH group of PHB. The band at 1278 cm^−1^ and 1379 cm^−1^ is sensitive to crystallinity of crystalline PHB. Figure 9a,b show a decrease in this peak with increasing take-up speeds, *i.e.*, increasing the take-up speed of electrospun fibers leads to a decrease the crystallinity of PHB and their blends. Increased take-up speed leads to a lower degree of crystallization; this is clear in the Figure 9c, with blends 1 and 2, at a constant take-up speed of 490 rpm, and blends 3 and 4 as casting film. It was found that the peak intensity at 1383 cm^−1^ was less in the case of the take-up speed at 490 rpm than in the casting film. The stretching peaks at 1452, 1379, 1223, and 2887 cm^−1^ correspond to the methyl group. A weak peak for the OH group of pure PHB appears between 3200 and 4000 cm^−1^, but the broad band of the OH group centered at 3400 cm^−1^ is attributed to the hydrogen bond between PHB and PANIs (blend 4 as a casting film). The intensity of OH increases with increasing PANIs content in the PHB matrix (blend 4 as a casting film). New peak appears at 1015 cm^−1^ for NH_2_ for PANIs. Hydrogen bonding happens between –NH_2_ or –OH group of PANIs and C=O of PHB and plasticizer.

## 4. Conclusions

Fibers of PHB and their blends have been fabricated by the electrospinning method using CF/DCM (75:25) and CF/DMF (90:10) as co-solvents. The addition of DMF demonstrated an increase in the solution conductivity after dissolving PHB in hot CF (90%) and then cold DMF (10%). It was found that the addition of cold DMF (10%) decreased the diameter of the fibers. The morphology, structure, and tensile properties of electrospun pure PHB and their blends were studied. SEM images showed that the average diameter of aligned electrospun fiber mats was smaller than those of random fibers. WAXD indicated that the crystallinity of the fibers decreased as the PANIs percentage increased compared to pure PHB, *i.e.*, electrospun fibers of blends have low crystallinity, making them suitable for many applications. Necking of electrospun fibers of pure PHB was not observed upon collection at the fixed plate (random) or rotating drum (aligned). Fibers of blends 1 and 2 showed multiple necking and were observed at a stretching rate of 390–970 rpm, but at a higher speed of 1100 rpm there is no necking, implying that the fiber is aligned. WAXD and FT-IR analyses indicate that, upon increasing the take-up speed of electrospun fibers, the crystalline structure of PHB deformed and the crystals are not well developed. The FT-IR shows that a new peak appears at 1015 cm^−1^ for NH_2_ and 3400 for OH group of PANIs. The tensile stress decreases from 29 MPa for pure PHB to 26 MPa for blend 2 after a take-up speed of 640 rpm, 16 MPa for blend 2 (random), and 10 MPa for blend 1 (random).

## Supplementary Materials

The following are available online at www.mdpi.com/2073-4360/8/3/97/s1, Electrospinning is a suitable method to produce fiber. The tool consists of a pumping device, small needle, higher voltage (DC) 0–40 kV and two types of collectors: a fixed plate (random) and moving drum (homemade). Figure S1: Electrospinning on the rotating drum in our lab (homemade); Figure S2: Electrospinning at a fixed target to obtain random fibers; Figure S3: Fiber rolling after electrospinning processes on the frame and the method to collects by the frame for the dumb bell shape.
