# Supplementary material for: Influence of Electrospinning Parameters on Fiber Diameter and Mechanical Properties of Poly(3-Hydroxybutyrate) (PHB) and Polyanilines (PANI) Blends
Source: Polymers (Basel). 2016 Mar 22;8(3):97. doi: 10.3390/polym8030097 (PMC6432519; doi:10.3390/polym8030097)
Supplement: Supplementary file 1 [file polymers-08-00097-s001.pdf]

Supplementary Material: Influence of Electrospinning Parameters on Fiber Diameter and Mechanical Properties of Poly(3-Hydroxybutyrate) (PHB) and Polyanilines (PANI) Blends

Ahmed M. El-hadi and F. Y. Al-Jabri

Electrospinning is a suitable method to produce fiber (MODEL NO BS-9000-2, Braintree Scientific, Braintree, MA, USA). The tool consists of a pumping device, 3 or 5 mL needle, a source of high voltage (DC), two types of collectors: a fixed plate (random) and moving drum (homemade, see Figure S1). Always cover the collector with new aluminum foil for each process. The idea of electrospinning is the use of high DC applied voltage (MODEL NO. ES60P-20W, Gamma High Voltage Research, Orlando, FL, USA) between a syringe needle and a grounded collector electrode, When the voltage reaches at the critical value, high voltage overcomes the surface tension of the solution inside the syringe, thereby creating a jet. The fiber collects and the solvent evaporates immediately.

Figure S2 shows the electrospun fiber on aluminum foil and the frame, which helps in the removal of fiber.

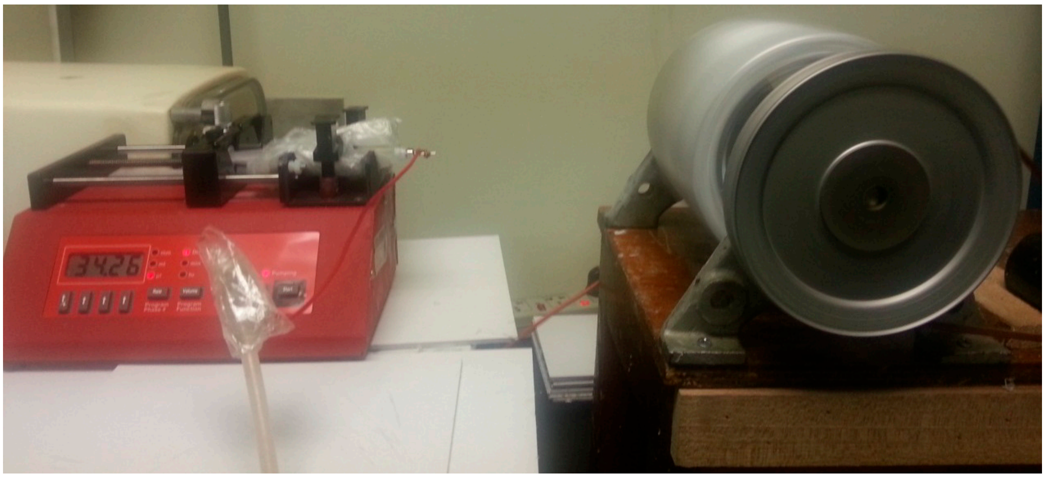

Figure S1. Electrospinning on the rotating drum in our lab (homemade).

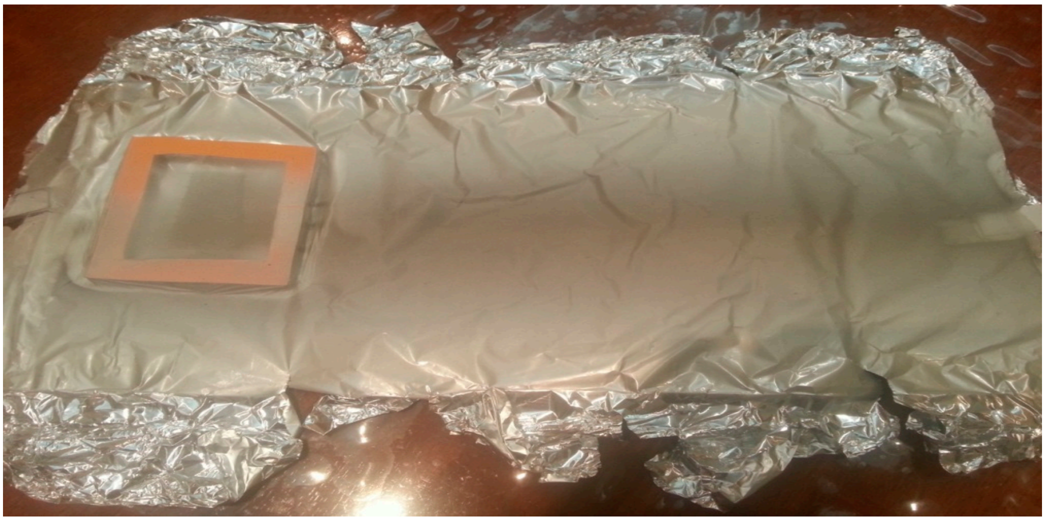

Figure S2. Electrospinning at a fixed target to obtain random fibers.

Generally, there are a number of parameters that affect the formation of fibers, such as flow rate, conductivity of viscosity and surface tension, the temperature of the solution, the applied voltage on the syringe needle, and humidity. Concentration of the solution is the most important parameter that is influenced in the formation of fibers without beads, which is associated with viscosity, the distance from the syringe needle to collector, needle diameter, and the type of solvent. At the beginning of the electrospinning process, the samples with chloroform (90%) are dissolved and heated using magnetic stirring, then let to cool for a few minutes. Then, after cooling, DMF (10%) is added with the plastic syringe (5 mL) and needle (1.3 mm), then put it in a pumping device. To remove the fiber from the collector for any measurements fiber is, at first, observed by POM and then its diameter determined by SEM. Square or rectangular frames of cork or paper can be used (see Figure S3).

The frame is placed before the start of the manufacture of fiber, which can be removed from the frames easily. If the fiber is spread on the collector and the frames rolled up to collect all of the fiber. It is a new idea to remove the fiber from aluminum surface easily. The square frames are used to measure by SEM and the rectangular frames are cut into a dumbbell shape (see Figure S3b,c) for tensile testing, where the frame size is suitable in the dumbbell shape.

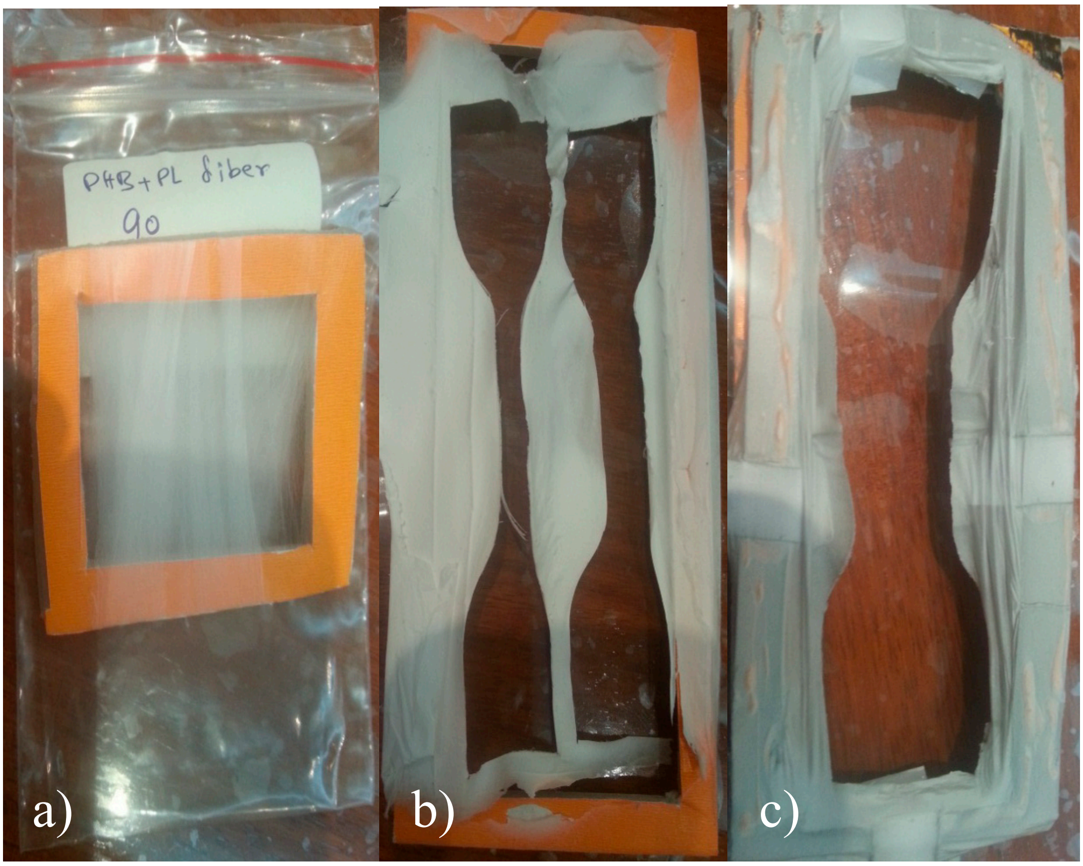

Figure S3. Fiber rolling after electrospinning processes on the frame and the method to collects by the frame for the dumb bell shape.

© 2016 by the authors; licensee MDPI, Basel, Switzerland. This article is an open access article distributed under the terms and conditions of the Creative Commons by Attribution (CC-BY) license (http://creativecommons.org/licenses/by/4.0/).
